# The Long-HER Study: Clinical and Molecular Analysis of Patients with HER2+ Advanced Breast Cancer Who Become Long-Term Survivors with Trastuzumab-Based Therapy

**DOI:** 10.1371/journal.pone.0109611

**Published:** 2014-10-20

**Authors:** Angelo Gámez-Pozo, Ramón M. Pérez Carrión, Luis Manso, Carmen Crespo, Cesar Mendiola, Rocío López-Vacas, Julia Berges-Soria, Isabel Álvarez López, Mireia Margeli, Juan L. Bayo Calero, Xavier González Farre, Ana Santaballa, Eva M. Ciruelos, Ruth Afonso, Juan Lao, Gustavo Catalán, José V. Álvarez Gallego, José Miramón López, Francisco J. Salvador Bofill, Manuel Ruiz Borrego, Enrique Espinosa, Juan A. Fresno Vara, Pilar Zamora

**Affiliations:** 1 Instituto de Genética Médica y Molecular (INGEMM) – IdiPAZ, Hospital La Paz, Madrid, Spain; 2 Medical Oncology Department, Hospital Quirón, Pozuelo de Alarcón, Madrid, Spain; 3 Medical Oncology Department, Hospital 12 de Octubre, Madrid, Spain; 4 Medical Oncology Department, Hospital Ramón y Cajal, Madrid, Spain; 5 Medical Oncology Department, Hospital de Donostia, San Sebastián, Pais Vasco, Spain; 6 Medical Oncology Department, Hospital Germans Trias i Pujol, Badalona, Barcelona, Spain; 7 Medical Oncology Department, Hospital Juan Ramón Jiménez, Huelva, Spain; 8 Medical Oncology Department, Hospital Clinic de Barcelona, Barcelona, Spain; 9 Medical Oncology Department, Hospital La Fe, Valencia, Spain; 10 Medical Oncology Department, Hospital Nuestra Señora de la Candelaria, Santa Cruz de Tenerife, Spain; 11 Medical Oncology Department, Hospital Miguel Servet, Zaragoza, Spain; 12 Medical Oncology Department, Hospital Son Llàtzer, Palma de Mallorca, Spain; 13 Medical Oncology Department, Complejo Hospitalario de Zamora, Zamora, Spain; 14 Medical Oncology Department, Hospital Serranía de Ronda, Ronda, Málaga, Spain; 15 Medical Oncology Department, Hospital Virgen de Valme, Sevilla, Spain; 16 Medical Oncology Department, Hospital Virgen del Rocío, Sevilla, Spain; 17 Medical Oncology Department, Hospital La Paz, Madrid, Spain; University of Pécs Medical School, Hungary

## Abstract

**Background:**

Trastuzumab improves survival outcomes in patients with HER2+ metastatic breast cancer. The Long-Her study was designed to identify clinical and molecular markers that could differentiate long-term survivors from patients having early progression after trastuzumab treatment.

**Methods:**

Data were collected from women with HER2-positive metastatic breast cancer treated with trastuzumab that experienced a response or stable disease during at least 3 years. Patients having a progression in the first year of therapy with trastuzumab were used as a control. Genes related with trastuzumab resistance were identified and investigated for network and gene functional interrelation. Models predicting poor response to trastuzumab were constructed and evaluated. Finally, a mutational status analysis of selected genes was performed in HER2 positive breast cancer samples.

**Results:**

103 patients were registered in the Long-HER study, of whom 71 had obtained a durable complete response. Median age was 58 years. Metastatic disease was diagnosed after a median of 24.7 months since primary diagnosis. Metastases were present in the liver (25%), lungs (25%), bones (23%) and soft tissues (23%), with 20% of patients having multiple locations of metastases. Median duration of response was 55 months. The molecular analysis included 35 patients from the group with complete response and 18 patients in a control poor-response group. Absence of trastuzumab as part of adjuvant therapy was the only clinical factor associated with long-term survival. Gene ontology analysis demonstrated that PI3K pathway was associated with poor response to trastuzumab-based therapy: tumours in the control group usually had four or five alterations in this pathway, whereas tumours in the Long-HER group had two alterations at most.

**Conclusions:**

Trastuzumab may provide a substantial long-term survival benefit in a selected group of patients. Whole genome expression analysis comparing long-term survivors vs. a control group predicted early progression after trastuzumab-based therapy. Multiple alterations in genes related to the PI3K-mTOR pathway seem to be required to confer resistance to this therapy.

## Introduction

Metastatic breast cancer is an incurable disease, with a median overall survival of approximately 3 years. However, patients’ outcome varies widely depending on a number of prognostic factors, such as visceral involvement, time from initial diagnosis, hormonal receptors and HER-2 status.

HER2 positive disease accounts for 15–20% of all cases and entails a poor prognosis [1]. Trastuzumab is a recombinant humanized monoclonal antibody that selectively targets the extracellular domain of the HER2 receptor. The prognosis of patients with HER2-positive metastatic breast cancer has dramatically changed since the advent of this drug [2], [3], [4], [5], [6]. Phase II and III studies have shown an advantage in response rate, disease-free survival and overall survival when the antibody is added to chemotherapy. Median overall survival was 25 months in the first reported trial of trastuzumab in advanced disease [2], and exceeded 37 months in a recent study [7]. In general, better results are seen in first-line as compared with subsequent lines of therapy [3].

Long-term survival can be achieved with trastuzumab and durable complete responses have occasionally been reported. Some of these women remain alive and disease-free after five to ten years from the diagnosis of metastases, which leads to hypothesize that cure could be possible in a small subset of patients. The Long-HER study was designed to analyse the clinical and molecular characteristics of HER2-positive advanced breast cancer in patients who obtained long-term responses with trastuzumab.

Microarray analysis is a widely used technology for studying gene expression on a global scale. Gene expression profiling is contributing important advances in clinical oncology, providing a basis for understanding the complex biology of tumours, improving the accuracy of disease diagnosis as well as disease prognosis, and providing tools to determine which targeted therapeutic agents are likely to be effective in the treatment of particular tumours. However, the requirement of frozen tissues for microarray experiments limits the clinical use of these gene signatures. Many laboratories are developing methods to assess gene expression profiling using formalin-fixed, paraffin-embedded (FFPE) samples [8], [9], [10].

In this work, we used genome-wide expression profiling of FFPE samples to study the molecular characteristics of breast cancers from patients with long-term complete response to trastuzumab. The aim of the study was to find clinical and molecular factors that would differentiate this population from patients developing treatment failure.

## Materials and Methods

### Ethics statement

Institutional approval from our ethical committee was obtained for the conduct of the study (Ethics Committee for Clinical Investigations, Hospital Universitario La Paz). The study was also approved by ethics committees from Área Sanitaria de Guipúzcoa and Hospital La Fe. Data were analyzed anonymously. Patients provided written consent so that their samples and clinical data could be used for investigational purposes.

### Study design

This was a multi-institutional retrospective study. Investigators searched their local databases to find patients with HER2 positive metastatic breast cancer who were long-term survivors. Participants had to fulfil all the following criteria: a confirmed diagnosis of metastatic breast cancer; HER2 positive disease, as determined by 3+ immunohistochemistry or positive fluorescence in situ hybridization (FISH); treatment with chemotherapy plus trastuzumab; stable disease or a sustained partial or complete response lasting at least 3 years; and availability of clinical data and tumour FFPE block. Clinical and radiological responses were assessed by local investigators using the Response Evaluation Criteria In Solid Tumors (RECIST) 1.1 (Eisenhower, EJC 2009; 45∶228). Treatment with anti-HER2 therapy other than trastuzumab was not allowed. Clinical characteristics were introduced in a database and the FFPE blocks were sent to a central laboratory. Molecular analysis would be performed in available samples from patients obtaining a complete response. A control group was also created with patients who had had a progression within the first 12 months of first-line trastuzumab-based therapy: molecular analysis was also performed in samples from this control group (poor-response group, hereafter). Patients from the control group came from a single institution (reference centre) and availability of tumour sample was required for these cases.

### RNA isolation and gene expression analysis

Selected FFPE tumour specimens were cut into serial sections with a thickness of 10 µm. RNA was then extracted with the RecoverAll Total Nucleic Acid Isolation Kit (Life Technologies). Since a significant part of RNA isolated from FFPE samples is not suitable for enzymatic reactions [11], we decided to use Pico Profiling [12], a novel method optimized for whole transcriptome amplification. Briefly, 100 ng RNA was subject to library synthesis and amplification for 23 cycles using WTA2 (Sigma Aldrich). 10 µg cDNA was subsequently fragmented by DNAseI and biotinylated by terminal transferase obtained from GeneChip Mapping 10Kv2 Assay Kit (Affymetrix). Hybridization, washing, staining and scanning of Affymetrix Human Genome U219 arrays were performed following the manufacturer’s recommendations. Scanned images (DAT files) were transformed into intensities (CEL files) by GCOS (Affymetrix). The raw intensity values were background corrected, log2 transformed, quartile normalized and summarized using the RMA algorithm in Genomics Suite 6.6 (Partek). Microarray data and clinical info are available at the Gene Expression Omnibus (GSE44272).

### Statistical analyses

Univariate analysis was used to assess the value of clinical and pathological variables: grade of differentiation, expression of hormonal receptors, size of the primary tumour, stage at diagnosis, number of metastatic sites, location of metastases, use of adjuvant chemotherapy, use of adjuvant trastuzumab, and time from initial diagnosis to relapse. Multivariate logistic regression was to be used with variables significant at univariate analysis.

Genes that were significantly up- or down-regulated between patient groups were identified using Significance Analysis of Microarrays (SAM) [13]. SAM assigns a score to each gene on the basis of a change in gene expression relative to the standard deviation of repeated measurements. For genes with scores greater than an adjustable threshold, SAM uses permutations of the repeated measurements to estimate the percentage of genes identified by chance – the false discovery rate (FDR). Analysis parameters (delta) were set to result in a FDR≤5%. Differentially expressed genes were investigated for network and gene functional interrelation using different bioinformatics tools [13]. Predictive models were built using a discriminant analysis approach in SPSS 16. SAM, hierarchical clustering analyses and mutations graphs were carried out with TIGR MeV v.4.7.4 [14]. Boxplots and univariant analyses were performed in GraphPad Prism 5.1.

### Gene ontology analyses

Gene ontology analyses including pathways, biological processes and molecular functions of genes related with trastuzumab response were performed in PANTHER [15]. Signalling pathway enrichment analyses were performed in SPEED [16].

### PI3K-mTOR pathway analysis

Genes related with PI3K pathway were selected from previous works [17], [18], [19]. For each gene all probes were selected and z-scored. Final expression values were the mean of all probes for each gene. Differences were assessed using the Mann Whitney test; significance was set at p<0.01.

### Mutations in Her2 breast cancer samples

To assess the mutational background of HER2 breast cancer samples, we used online available information about the mutations identified in 30 HER2 positive samples previously analysed [20]. We selected all mutations and copy number variants in these samples, then we investigated which genes differentially expressed in our study had any mutation or copy number variant. Finally, we performed the same analysis with genes from the PI3K-mTOR pathways.

## Results

### Clinical data

A total of 103 long-term survivors were identified, of whom 71 had achieved a durable complete response. Median age was 58 years. Median duration of response for the group of 103 survivors was 55 months (35–127) and median overall survival was 59 months (36–130). Two thirds of the tumours were poorly differentiated and 51% did not express oestrogen or progesterone receptors. HER2 status had been determined by immunohistochemistry in 98% of cases and by FISH in 21%. At the time of initial diagnosis, 21% of patients had received neoadjuvant chemotherapy and 70% had received adjuvant chemotherapy. In all but 11 patients, adjuvant or neoadjuvant chemotherapy was anthracycline-based. Twenty-eight percent of patients had received adjuvant hormonal therapy. Four patients (8%) had been treated with adjuvant trastuzumab. Table 1 summarises clinical features.

**Table 1:** Summary of patients’ characteristics.

	All Long-HER, n = 103	Complete responders n = 71	Complete responders with molecular analysis, n = 35	Control poor-response group, n = 18
**Median age (years)**	58 (36–87)	58 (40–87)	59 (40–87)	57 (41–83)
**Hormone receptors +**	49%	47%	49%	56%
**Location of mets.**				
Liver	14 (13%)	18 (25%)	6 (11%)	2 (11%)
Lung	24 (23%)	18 (25%)	15 (43%)	6 (33%)
Bones	15 (14%)	16 (23%)	11 (31%)	3 (17%)
Soft tissues	6 (6%)	16 (23%)	10 (29%)	6 (33%)
Multiple sites	32 (31%)	14 (20%)	8 (23%)	6 (33%)
**Adjuvant or neoadjuvant therapy**	90 (91%)	66 (94%)	32 (93%)	14 (78%)
**First-line therapy**				
Anthracycline-based	9 (9%)	4 (6%)	2 (6%)	0
Taxane-based	41 (40%)	35 (49%)	22 (63%)	13 (72%)
Anthracycline+taxane	34 (33%)	19 (27%)	8 (23%)	1 (6%)
Other chemotherapy	11 (10%)	8 (11%)	3 (8%)	4 (22%)
Hormonal therapy	8 (8%)	5 (7%)	0	0
Trastuzumab duration	61 months (6–148)	62 months (12–138)	62 months (12–138)	10 months (3–15)

The whole population of long-term responders included 103 patients, of who 71 achieved a complete response. Molecular analysis was performed in 35 out of 71 patients with complete response.

All 103 patients received trastuzumab as first-line therapy. This treatment began between the years 1999 and 2007. Ninety-five patients also received concomitant chemotherapy, which included a taxane in 65 (63%) and an anthracycline in 43 (42%). Four patients had to discontinue chemotherapy due to toxic events. Forty-two patients were treated with a hormonal agent, either initially (8 patients) or after the achievement of a complete response with chemotherapy (34 patients). Initial hormonal therapy consisted in letrozol in 4 patients, exemestane in 2 patients and tamoxifen in 2 patients. As inclusion criteria required treatment with chemotherapy, these 8 patients initially treated with a hormonal agent were not accepted for the molecular analysis. Fifty-three patients remain in remission and 48 are still receiving trastuzumab-based therapy; fifty patients have stable disease and 45 of them also continue on trastuzumab. In the whole group of long-term survivors with complete response (n = 71), the liver and the lungs were the most common metastatic sites (25% each), followed by the bones, skin and lymph nodes (23% each). Twenty percent of patients had multiple affected sites. The control group included 18 patients coming from one single centre. These patients had begun first-line therapy with trastuzumab between the years 2005 and 2007. All these patients had clinical data and tumour samples available.

Clinical factors such as grade of differentiation, number of metastatic sites or the location of metastases did not correlate with long-term survival. There were five patients with brain metastasis among survivors: these five patients received local treatment (surgery for isolated lesions plus radiation therapy) in addition to systemic therapy. Only 6 out of 103 patients presented with metastasis in soft-tissues exclusively. Only four of the long-term survivors had received adjuvant trastuzumab. This was the only clinical factor associated with prolonged survival.

### Tissue specimens and patients’ characteristics for molecular analyses

Samples for the molecular study were selected from the 71 patients achieving a complete response. The molecular study also included a control group coming from patients who had a poor response after trastuzumab-based therapy. In patients with complete response, median duration of response was 62 months (35–127) and the median overall survival 67 months (36–130). In the control group, median duration of response was 6 months (2–12) and the median overall survival 33 months (15–124). Sixty-one FFPE samples with annotated clinical information were selected, 53 of which had enough RNA to perform hybridization. Among these 53 samples, 35 samples corresponded to long-term responders and 18 to the control group. Table 1 shows the clinical features of all Long-HER survivors, Long-HER patients achieving a complete remission, Long-HER patients selected for molecular analysis and the control group. There were no major differences among these categories, although the 71 Long-HER survivors had a lower incidence of liver and lung metastases as compared with the other groups.

### Genes related with response to trastuzumab

**Figure 1:**
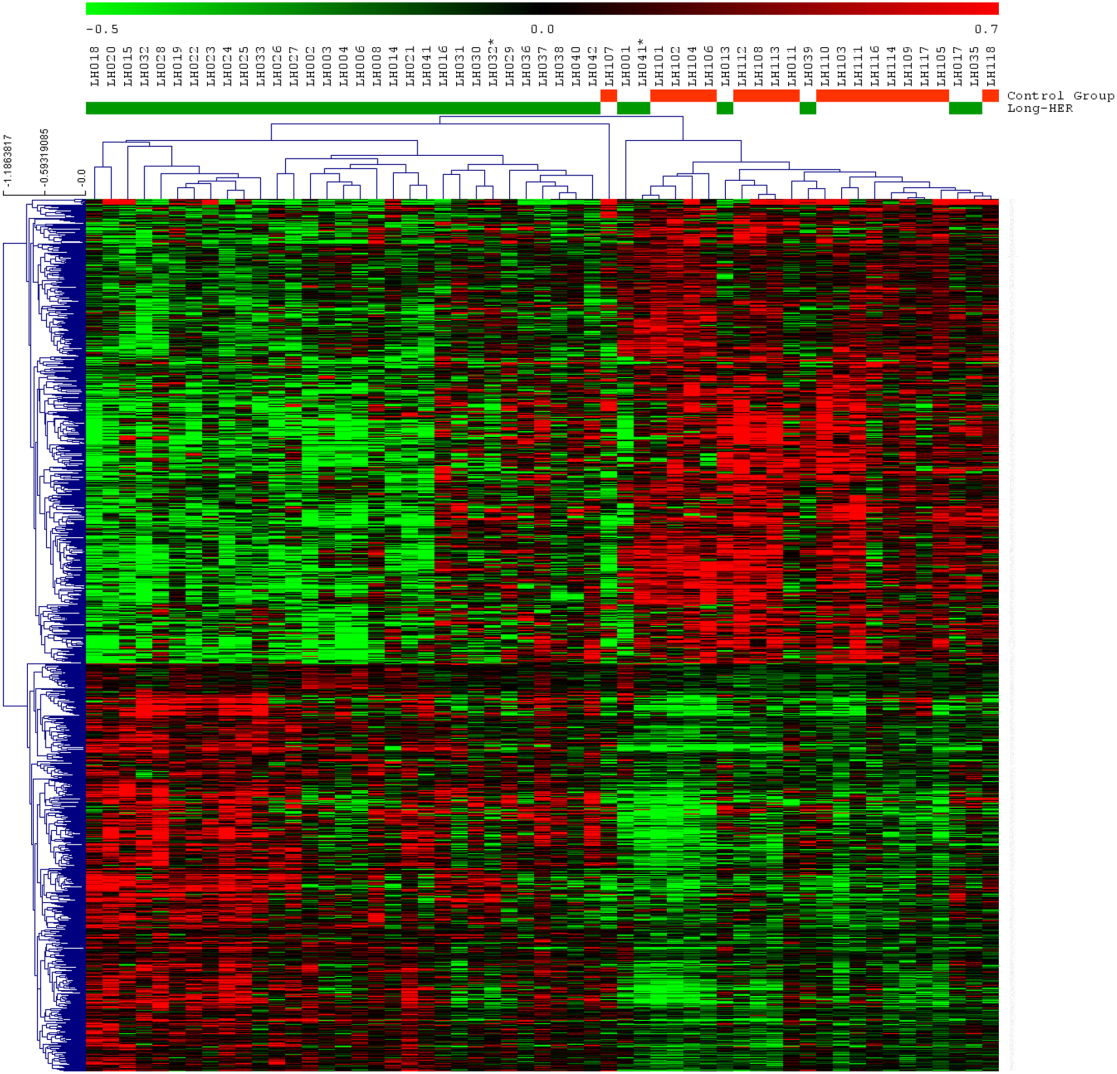
Supervised hierarchical clustering for all samples using 1052 differentially expressed probesets identified using SAM. Each row represents a probeset and each column a sample. Green bars indicate samples from patients in Long-HER group, red bars indicate samples from short-term responders. There are two samples duplicated, * account for metastasis samples.

**Figure 2:**
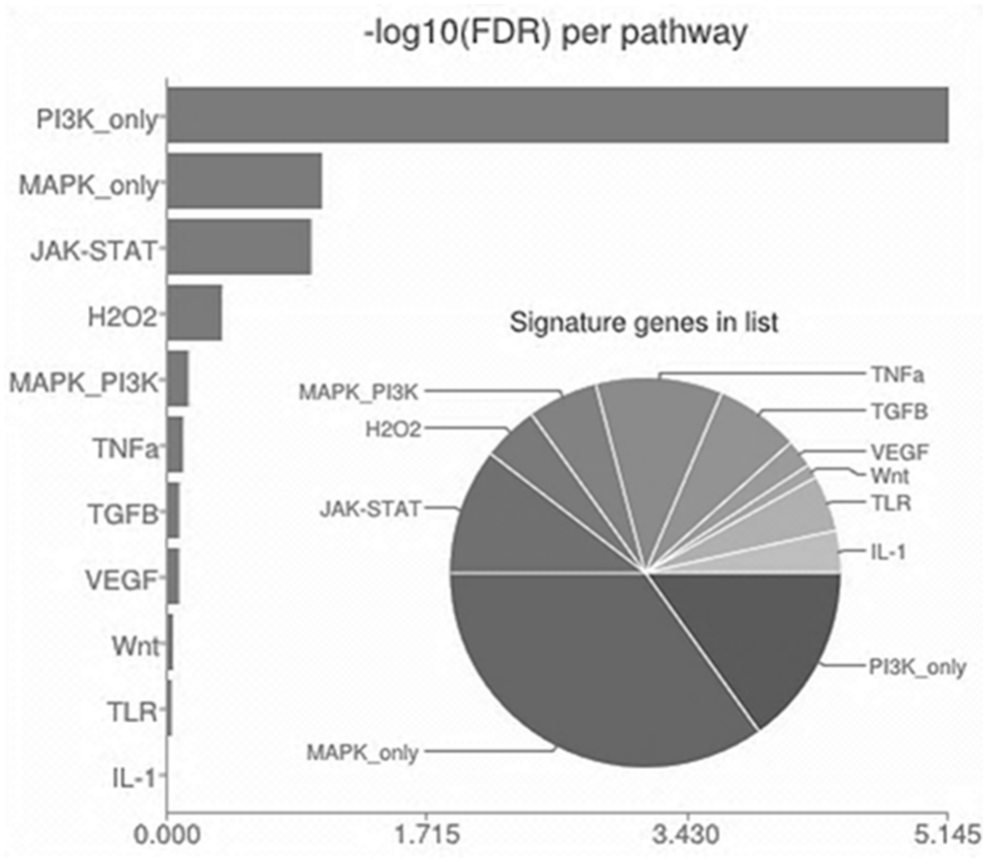
Signaling pathway annotation enrichment analysis for 858 genes related with trastuzumab resistance. This analysis was performed using SPEED software. PI3K pathway appeared as the most relevant pathway in relation with trastuzumab resistance.

Comparison of the control group and Long-HER group using SAM showed significant differences in the expression of 1052 probe sets representing 858 genes. 560 probe sets were up-regulated, and 492 probe sets were down-regulated in the control group compared to the Long-HER group (Figure 1 and Table S1). In order to identify those pathways associated with the response to trastuzumab, functional categories of 858 differentially expressed genes were identified using gene ontology analysis. Statistically significant categories included a large number of genes involved in hypoxia response via HIF activation, EGF receptor signalling pathway, PI3 kinase pathway, apoptosis signalling pathway and p53 pathway (Figure S1). Signalling pathway enrichment analyses revealed the PI3K pathway as the most strongly associated with response to trastuzumab, as shown in Figure 2.

### PI3K-mTOR pathway analysis

**Figure 3:**
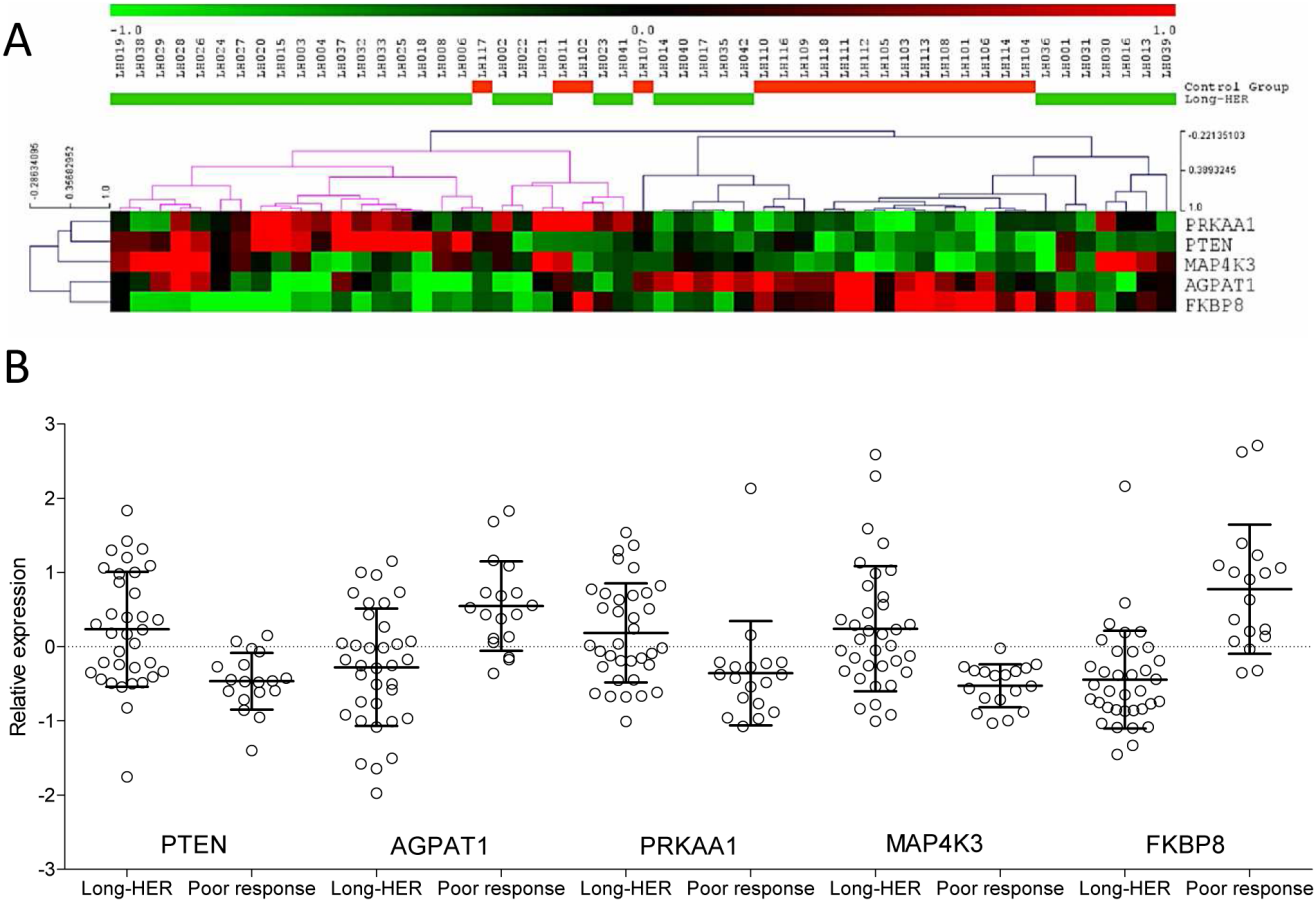
PI3K-mTOR pathway analyses. A) Supervised hierarchical clustering for all samples using five genes from the PI3K-mTOR pathway that are differentially expressed between Long-HER and short-term responders to trastuzumab samples. B) Distribution of the normalized expression of these genes.

To elucidate the mechanisms responsible for trastuzumab resistance, 338 probes comprising 97 genes related to the PI3K-mTOR pathway were evaluated. Fourteen genes were differentially expressed between the control group and the Long-HER group (Mann-Whitney p<0.01). Five of these genes -*PTEN, AGPAT1, PRKAA1, FKBP8* and *MAP4K3*- were found to act upstream of *mTOR* complexes, and could modulate *mTOR* signalling. Expression values for these genes are shown in Figure 3. Most patients in the control group had low expression of *PTEN, MAP4K3* and *PRKAA1*, and high expression of *AGPAT1* and *FKBP8*. However, these features appeared as isolated events in the Long-HER group (Figure 3). A predictive model for trastuzumab response, including *PTEN, AGPAT1, PRKAA1 FKBP8* and *MAP4K3* gene expression, was built using a discriminant analysis approach. The model classified correctly more than 81% of the samples in the Leave-one-out cross validation and presents an area under the curve of 0.938.

### Analysis of the mutational state in HER2-positive breast cancer samples

**Figure 4:**
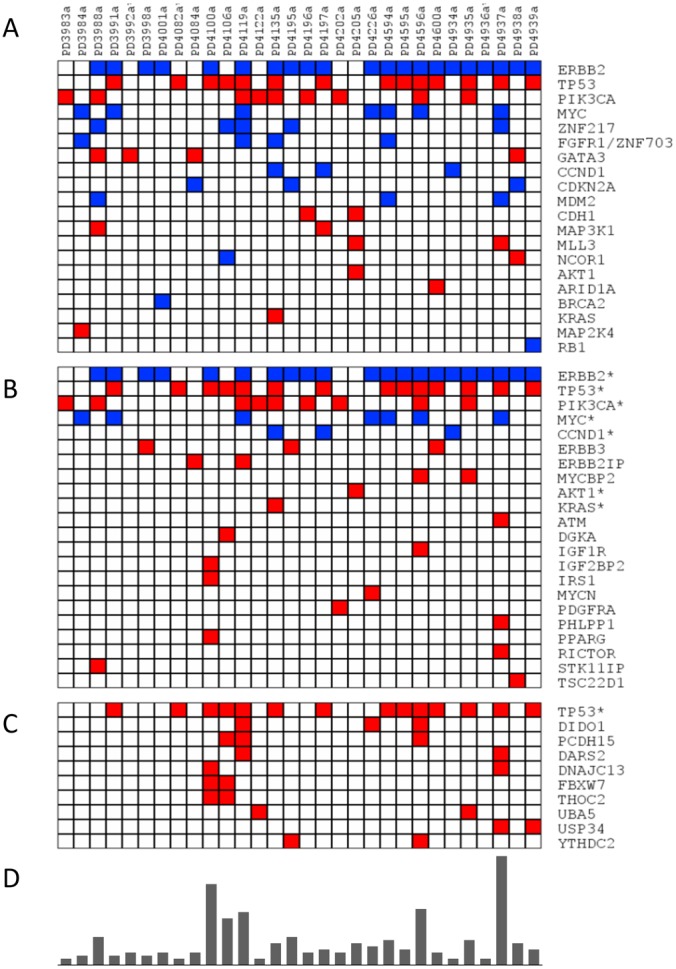
Mutational status of HER2+ patients. Distribution of point mutations (red) and copy number variants (blue) in A) genes considered as carriers of cancer driven mutations by Stephens and co-workers, B) genes from the PI3k-mTOR pathway, and C) genes related with trastuzumab resistance (only genes with two of more mutation events are showed, full report is provided as Additional Figure 1). D) Number of point mutations and copy number variants detected in each patient. *This gene is also considered as carrier of cancer driven mutations.

Using online available data from another study, we assessed the mutational status of the 858 genes related to trastuzumab resistance found in our study, with particular attention to the 97 genes related to the PI3K-mTOR pathway. To do this, online data from a previously described analysis of mutations and copy number variants in 30 HER2-positive tumours were used [20]. We compiled 2,229 point mutations across the exome and 101 copy number variations considered by the authors as cancer drive mutations. We found 143 point mutations distributed in 114 out of 858 trastuzumab resistance related genes (13%). The number of mutations in each sample varied widely, from 2 to 35 (mean 7.6). Only 10 of these mutations appeared in two of more patients (Figure 4). *TP53* presented the highest mutational rate (14/30 samples, 47%), followed by *PIK3CA* (30%), *ERBB3, DIDO1* and *PCDH15* (10% each). There were also 46 point mutations in 17 out of the 97 genes (17%) in the PI3K-mTOR pathway. Additionally, we found 51 copy number variants in 10 regions containing genes considered as drivers of cancer. *ERBB2* region was amplified in 21 out of the 30 samples (70%), whereas 7/30 (23%) presented MYC amplification (Figure 4). Interestingly, point mutations or copy number variants of *PTEN, AGPAT1, PRKAA1, FKBP8* and *MAP4K3* were not present in these 30 HER2 positive patient samples.

## Discussion

Long-term survival without evidence of disease is possible in a small subgroup of patients with metastatic HER2-positive breast cancer. The Long-HER study analysed the clinical and biological characteristics of such patients treated with trastuzumab-based therapy and compared the results with those obtained in a similar series of patients who had experienced disease progression within the first year of treatment.

Clinical factors did not correlate with long-term survival. The presence of brain metastases in five patients with long-term survival implies that central nervous system spread does not preclude long-term survival. Only 6 out of 103 patients presented with metastasis in soft-tissues exclusively, meaning that the vast majority of our study population had visceral dissemination.

Long-term survivors had not received adjuvant trastuzumab because the drug was not used in this indication at the time of initial diagnosis. This was the only clinical factor associated with prolonged survival and suggests the existence of tumours highly sensitive to trastuzumab that could obtain prolonged remissions regardless of the time of drug administration. As trastuzumab is now part of the standard adjuvant therapy for HER2-positive breast cancer, the population analysed in the Long-HER study will be increasingly difficult to find because such patients are cured by adjuvant trastuzumab. An Irish-Italian consortium has recently reported on 13 patients who achieved durable complete responses with trastuzumab-based therapy [21]. These patients had received at least two years of treatment with the monoclonal antibody, whereas many of our patients have continued on therapy beyond 5 years. Other recent studies have reported similar series of long-term survivors among patients treated with trastuzumab [22], [23], [24].

Tumours in the Long-HER group did not show a uniform expression gene profile. In other words, molecular identification of this subset of patients with excellent prognosis remains an elusive goal. However, tumours in the control group, i.e., those having early progression to trastuzumab-based therapies, were clearly identified in the present study. The Long-HER study serves as a proof-of-principle that whole genome expression analysis on FFPE samples can provide novel mechanistic insights related to drug resistance/sensitivity. Anthracyclines were less used in the control group as compared with the Long-HER in first line, which could have influenced the rate of initial remissions. However, we did not find a significant difference in the Long-HER group according to the type of chemotherapy. Also, the molecular analysis was not influenced by the type of chemotherapy because samples were taken before initiation of first-line therapy.

Mechanisms of resistance to trastuzumab have been categorised in three groups: structural mutations in HER2 protein, increased expression or activation of other tyrosine kinase receptors (such as insulin-like growth factor receptor), or intracellular alterations in HER2 downstream signalling [25], [26]. This latter mechanism includes PTEN deficiency [27], SRC activation [28] and PI3K/AKT activation [29]. In our study, genes related to the PI3K pathway appeared most strongly associated with poor response, although other alterations were also found. These results suggest that resistance to trastuzumab does not depend on one or a limited number of mutations, but rather on a variety of changes in involved pathways. A recent review on genomic analysis indicates that the polygenic nature of drug resistance, as well as tumour heterogeneity, constitute important challenges in the development of response predictors [30]. For this reason, gene or protein profiles may provide a more realistic approach than single marker determination to assess resistance.

SAM analyses identified 858 genes related to trastuzumab resistance in our series. Gene ontology and signalling pathway enrichment analyses pointed out the relation between PI3K-mTOR signalling pathway and trastuzumb resistance. PI3K-mTOR signalling pathway analyses showed that tumours from patients who did not obtain prolonged remissions (control group) usually had loss of expression in *PTEN, PRKAA1* and *MAP3K4*, as well as overexpression in *AGPAT1* and *FKBP8*. On the contrary, tumours from the Long-HER group could present with none, one or two of these molecular alterations, which suggests that more than two events (either these or other with similar consequences) are needed to confer resistance to trastuzumab.

**Figure 5:**
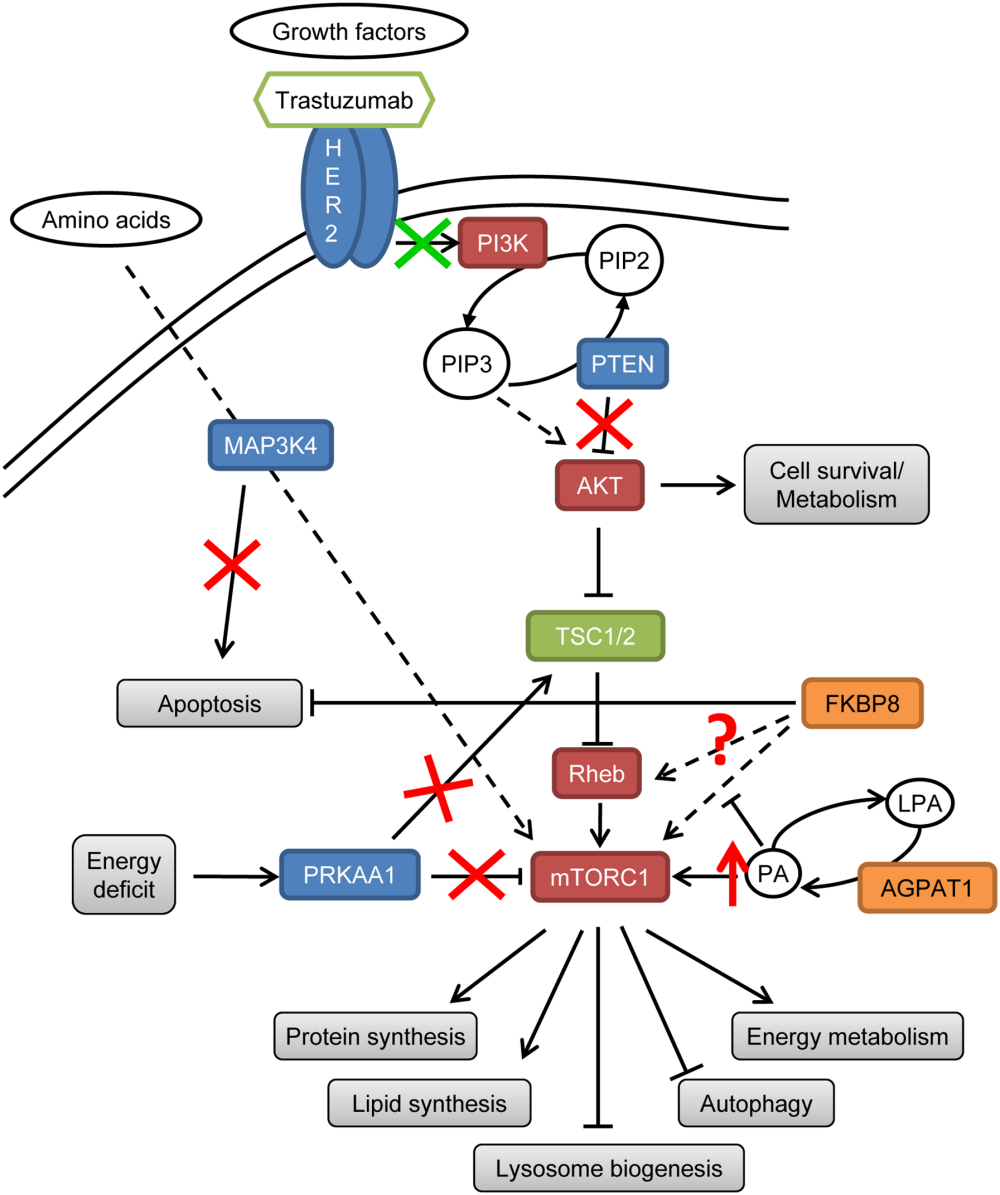
Proposed model of trastuzumab resistance in short-term responders due to Akt/mTOR activation and apoptosis inhibition. Genes in blue are downregulated, genes in orange are upregulated, genes in red favour Akt/mTOR pathway activation and genes in green decrease Akt/mTOR pathway activation. Red signs are new disrupting elements identified. Green arrow indicates the step regulated by trastuzumab.

Loss of *PTEN* expression has been previously associated with resistance to trastuzumab [31]. Active PRKAA1 directly phosphorylates and enhances the ability of tuberin to inhibit mTOR signalling [32]. Loss of PRKAA1 can lead to an increased mTORC1 activity independently of the energetic status of the cell [33]. MAP4K3 plays an important role in the regulation of mTOR signalling in response to amino acids presence and its loss could disturb cell sensitivity to nutrient absence [34]. MAP4K3 loss can decrease BAX activation, inactivating the apoptosis pathway [35]. AGPAT1 catalyses the conversion of lysophosphatidic acid to phosphatidic acid (PA) [36] and over-expression of AGPAT1 can increase PA in the cell, constitutively activating mTOR [37]. The molecular interaction of Bcl-2 with FKBP8 potentiates the biological function of Bcl-2 and contributes to tumorigenesis and chemoresistance [38]. Additionally, it has been proposed that FKBP8 mediates the activation of mTORC1 by Rheb [39], [40], [41]. Although recent reports dispute this idea [42], [43], it is clear that FKBP8 binds both Rheb and mTORC1, and this interaction is disrupted in the presence of PA [44]. All these gene alterations lead to Akt/mTOR activation and apoptosis inhibition (Figure 5), and could explain the lack of response to trastuzumab in the control group.

Our analysis of 858 genes in a whole-genome mutation database [20] suggests that a common mutation profile does not exist for this population because the incidence of mutations varies widely. Answering clinical questions by performing mutational analysis may be difficult, but expression profiling of either mRNA or proteins could provide useful insights. In our study, the mechanistic analysis of the PI3K-mTOR pathway allowed the identification of some potential key gene expression alterations in a selected population of patients.

Previous studies have shown that PI3K activation and loss of PTEN predict trastuzumab resistance [27], [31], [45], [46]. However, an mTOR inhibitor has recently shown a modest benefit in patients progressing upon trastuzumab, which suggests that further investigation is required before taking these findings to the clinic [47], [48]. These trials include patients who have had a progression with trastuzumab-based therapy, but the possibility to identify tumours that will likely be resistant to trastuzumab could be important for the development of therapeutic strategies in earlier stages of disease. Patients less likely to be cured with adjuvant trastuzumab, for instance, would be ideal candidates to participate in clinical trials with new anti-HER2 drugs.

Our study has some limitations. First, patients received a variety of chemotherapy agents along with trastuzumab. A taxane was used in most cases, but no clinical or molecular differences were detected in patients treated with other drugs. This suggests that it is the monoclonal antibody and not its companion what determines prognosis. On the other hand, molecular findings must be interpreted with caution because we analysed many genes and sample size was small. Also, the limited sample size does not allow getting further insight into the molecular pathways involved in the response to trastuzumab. Finally, a proportion of samples in the Long-HER group were not suitable for molecular analysis. We tried to recruit as many patients as possible to gather clinical information, but unfortunately many of corresponding samples were not available or were in poor condition for molecular analysis. This could have produced an uncontrolled bias in the results of the molecular study. On the contrary, patients in the control group were selected from a reference centre with optimal storing conditions and whit the requirement that tumour samples would be available. Validation in an independent series of patients is obviously required. However, very few patients achieve long-term survival, as demonstrated in our series and other published series, so a formal validation may be difficult to perform.

In summary, long-term survival is possible in a selected population of patients with advanced HER2-positive breast cancer. Whole genome expression analysis comparing long-term survivors vs. a control group was able to predict progression to trastuzumab-based therapy. Multiple alterations in genes related to the AKT-mTOR pathway seem to be required to confer resistance to this drug. The validation of a poor-response profile would allow tailoring therapy in future clinical trials.

## Supporting Information

Figure S1 Gene Ontology Analyses. Gene ontology analyses including A) pathways, B) biological process and C) molecular functions of the 858 genes related with trastuzumab response were performed using PANTHER. All categories represented are differentially represented when compared with the *Homo sapiens* reference list (P<0.05).
(TIF)

Table S1 Expression values of 1052 probe sets differentially expressed between Long-Her and control group. ER: Estrogen receptor status; PGr: Progesterone receptor status; PFS: Progression free survival; M: Metastases; N: lymph node status; T: Tumor size. Clinical criteria are provided according to TNM classification. (http://www.cancer.gov/cancertopics/pdq/treatment/breast/healthprofessional/page3).
(TXT)
