# Supplementary figures and images for: The Long-HER Study: Clinical and Molecular Analysis of Patients with HER2+ Advanced Breast Cancer Who Become Long-Term Survivors with Trastuzumab-Based Therapy
Source: PLoS One. 2014 Oct 20;9(10):e109611. doi: 10.1371/journal.pone.0109611 (PMC4203741; doi:10.1371/journal.pone.0109611)

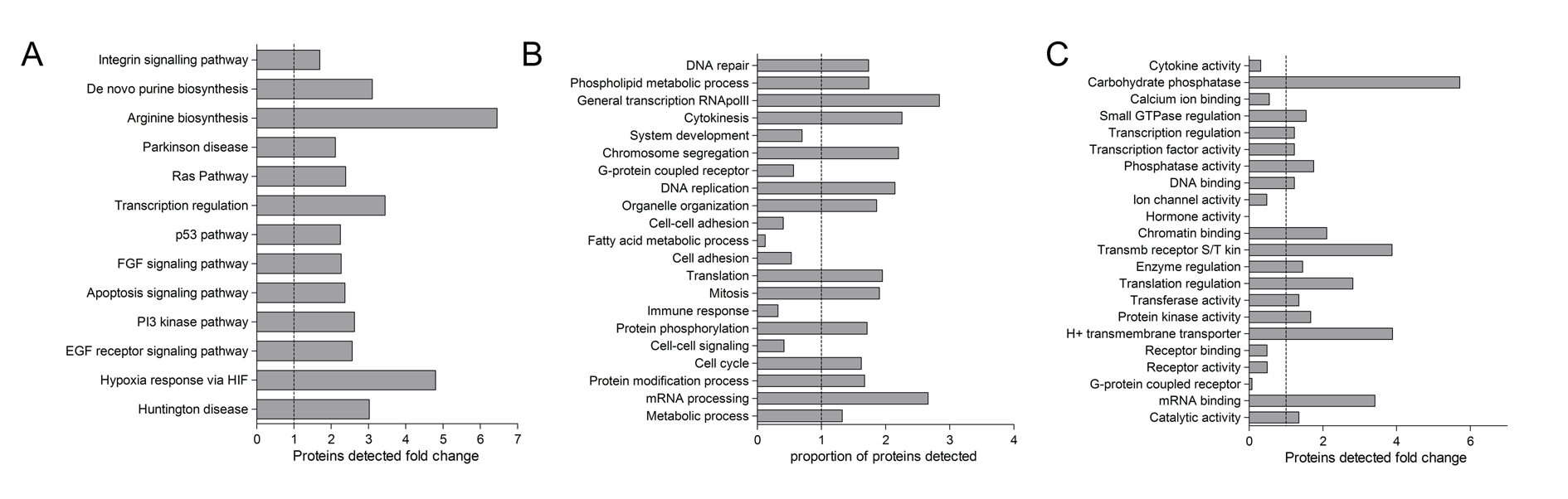

Supplement: Figure S1 — Gene Ontology Analyses. Gene ontology analyses including A) pathways, B) biological process and C) molecular functions of the 858 genes related with trastuzumab response were performed using PANTHER. All categories represented are differentially represented when compared with the Homo sapiens reference list (P<0.05). (TIF) [file pone.0109611.s001.tif]
